# Translation and Validation of a Chinese Version of the Mindfulness in Parenting Questionnaire (MIPQ)

**DOI:** 10.3389/fpsyg.2019.01847

**Published:** 2019-08-14

**Authors:** Lei Wu, Heather Buchanan, Yaping Zhao, Ping Wang, Zhao Zhan, Boyao Zhao, Bijuan Fan

**Affiliations:** ^1^Key Research Base of Humanities and Social Sciences of the Ministry of Education, Academy of Psychology and Behavior, Tianjin Normal University, Tianjin, China; ^2^Division of Rehabilitation and Ageing, School of Medicine, University of Nottingham, Nottingham, United Kingdom; ^3^School of Education, Xinyang University, Xinyang, China; ^4^Wuxi Tianyi Experimental School, Wuxi, China

**Keywords:** Mindfulness in Parenting Questionnaire, reliability, validity, psychometric, China

## Abstract

**Objective:**

To translate the Mindfulness in Parenting Questionnaire (MIPQ) into Standard Mandarin and then explore the reliability and validity of this newly translated measure in a large sample of Chinese middle school parents.

**Methods:**

We translated the MIPQ using the forward-backward method and pilot tested it on a sample of parents of adolescents (aged 12–16 years) in China. Following minor modifications, 1057 Chinese parents (Mothers or Fathers) in two middle schools (one in the North and one in the South of China) completed the translated Chinese Mindfulness in Parenting Questionnaire (C-MIPQ). To determine test-retest reliability 121 participants completed the C-MIPQ again 2 weeks later. In order to test convergent validity, 395 participants completed the Mindful Attention and Awareness Scale (MAAS), Parenting Styles and Dimensions Questionnaire-Short Version (PSDQ-short) and the Interpersonal Mindfulness in Parenting Scale (IM-P). The Chinese Perceived Stress Scale (CPSS), Beck Depression Scale (BDI-13), and socioeconomic status (SES) were completed in order to test discriminant validity.

**Results:**

Confirmatory factor analysis showed that the two-factor model indicated in the original study was a good fit. The total score of the scale and the scores of the two dimensions (‘Mindful discipline’ and ‘Being in the moment with the child’) were significantly positively correlated with the total score of the MAAS, IM-P and the authoritative parenting style in the PSDQ-short, demonstrating convergent validity. Discriminant validity was established as there was no difference in C-MIPQ sub-scale scores across any of the SES variables except for levels of education (parents with higher education had higher scores on the ‘being in the moment with the child’ subscale). In addition, the C-MIPQ was negatively related to stress and depression. Cronbach’s alpha of the total scale was 0.93 (and 0.88, 0.89 for the two dimensions of the C-MIPQ) indicating excellent internal consistency. Test-retest reliability was good (intra-class correlation of 0.83).

**Conclusion:**

This study is the first step toward establishing the psychometric properties of the C-MIPQ for measuring mindful parenting in parents of adolescents aged 12–16 years; additional studies will be needed in order to test this further.

## Introduction

Adolescence is as an important stage of development with dramatic changes in cognitive, physical and psychological functions. Good mental health at this time is particularly important. However, a social survey on the mental health status of adolescents in China’s nine provinces showed that twelve percent of adolescents had poor mental health (Research Group for the Survey on Chinese Adolescents’ Mental Health, 2013). Adolescent mental health is affected by multiple factors such as family, school and society. The family is the primary place for the socialization of young people and is important for individual growth and development. From the investigation of factors affecting the mental health of adolescents, it has been found that parenting is one of the main influences on the mental health of adolescents (Wolfradt et al., 2003; Gong and Paulson, 2017; Gouze et al., 2017).

Parenting style refers to the general attitudes and behaviors of parents raising and educating their children and the values of child development (Darling and Steinberg, 1993). There are two main kinds of research on parenting style. The first one is to categorize parenting behaviors into several types. For example, Baumrind (1967, 1978, 1991) proposed four types of parenting (authoritative, authoritarian, permissive/indulgent, and uninvolved). As an example of one of these parenting types, authoritarian parenting means parents have strict rules for their children and they exert behavioral control over them; this can lead to negative emotions for adolescents including depression, sadness, and anger (Konopka et al., 2018). The second kind of parenting research focuses more on specific parenting behaviors, rather than categorizing into types. For example, Perris et al. (1980) proposed 15 parenting behaviors including deprivation, punishment, tolerance, and encouragement.

In recent years, the concept of ‘mindfulness’ has also been introduced as a style of parenting. ‘Mindful parenting’ refers to a non-judgmental and open parenting style. Duncan et al. (2009) proposed that mindful parenting should include five aspects: (1) listening with full attention to the child (2) non-judgmental acceptance of the self and the child (3) emotional awareness of the self and the child (4) self-regulation in the parenting relationship and (5) compassion for the self as a parent and for the child. Duncan et al. highlighted that all of these aspects can affect the well-being, parent-child relationship and subsequent psychopathological symptoms of parents and adolescents.

Mindful parenting is often associated with other positive parenting behaviors. For example, Benton et al. (2019) found that mindful parenting is related to maternal sensitivity. High sensitivity helps parents better understand and accept their children and reduces excessive emotional reactions in the parent-child interaction (Biringen et al., 2014). In addition, it has been found that parents with high levels of mindful parenting often adopt other positive parenting practices, e.g., maintaining consistency in parenting (Williams and Wahler, 2010; Geurtzen et al., 2015). Moreover, Parent et al. (2016) found that parents with high levels of mindfulness treated their children with warmth and used positive reinforcement.

Higher mindful parenting is also associated with better mental health of parents. For example, mindful parenting interventions can help alleviate symptoms of anxiety and depression (Corthorn and Milicic, 2016; Parent et al., 2016) and negative behaviors (e.g., antagonistic behavior) of parents (Bögels and Restifo, 2014). In addition, researchers have found that psychopathological symptoms for parents decreased after taking part in a mindful parenting intervention (Meppelink et al., 2016).

Mindful parenting has a positive influence on children’s development too. For example, high mindful parenting can positively influence the mental health of adolescents (Parent et al., 2010; Williams and Wahler, 2010; Geurtzen et al., 2015; Tak et al., 2015; Parent et al., 2016; Carlson et al., 2017). Studies have shown that parents with high levels of mindful parenting have less controlling behavior toward their children, which is known to lower self-esteem of children (Lippold et al., 2015). Mindful parenting can also help to build a good parent-child relationship (Lippold et al., 2015) and have a positive impact on the child’s overall quality of life (Dehkordian et al., 2017).

### Mindful Parenting Scales

At present, there are two published mindful parenting scales. Duncan (2007) developed the first scale for measuring mindful parenting. It is the Interpersonal Mindfulness in Parenting (IM-P) scale. The IM-P scale was developed for parents of young people aged between 10 and 14 years. It initially had only 10 items. More recently, the IM-P was expanded to 31 items and translated into Dutch (De Bruin et al., 2012). In this study, the IM-P was validated in a sample of Dutch mothers of adolescents (12–15 years). The findings resulted in 29-items with a six-factor structure. However, this 29-item IM-P only applies to measuring mother’s mindful parenting. In addition, both the shorter and longer versions of IM-P are limited in the applicable age range (10-item for the parents of individuals aged 10–14 years and the longer version for parents of individuals of 12–15 years). Thus, a measure which is applicable to both parents, and suitable for a wider age range of children and young people was warranted.

McCaffrey et al. (2017) developed a measure of mindful parenting for both mothers and fathers of children and adolescents (the Mindfulness in Parenting Questionnaire; MIPQ) by using modern test theory approaches. They conducted a validation study in the USA on 203 parents of children aged 2–16 years. Results showed a two-factor measure of mindful parenting. The first factor is ‘being in the moment with the child,’ which reflects a child-focused facet of mindful parenting. It comprises present-centered attention to the child, empathic understanding of the child, and acceptance of the child. The second factor is ‘Mindful discipline’ which reflects the parent-focused facet. It includes non-reactivity in parenting, parental awareness of parenting, and goal-focused parenting. The two-factor structure of the MIPQ maps well onto the five aspects of mindful parenting proposed by Duncan et al. (2009). The factor ‘being in the moment with the child’ in the MIPQ corresponds to ‘listening with full attention,’ ‘emotional awareness,’ and ‘compassion’ within Duncan’s five aspects. The factor ‘mindful discipline’ corresponds to ‘non-judgmental acceptance’ and ‘self-regulation in parenting relationship.’ The six subscales of the IM-P found by De Bruin et al. (2012) also correspond to the five aspects of mindful parenting, with ‘emotional awareness of the self and the child’ from Duncan et al. (2009) splitting into two subscales ‘emotional awareness of the self’ and ‘emotional awareness of the child’ in the IM-P. Although the two-factor solution of MIPQ and the six subscales of IM-P can both represent the five aspects of mindful parenting well, the two-factor solution of the MIPQ is arguably more efficient and comprehensive than the four-factor structure of IM-P. Some of the subscales of the IM-P overlap with one another and some sub-scales only include items from either a parent-oriented or child-oriented facet. For example, in the subscale of ‘non-judgmental acceptance’ on the IM-P, all items are parent-oriented. The two subscales of the MIPQ on the other hand include items from both parent and child-oriented facets. Thus, we consider the MIPQ the scale of choice when assessing mindful parenting.

### Mindful Parenting Research in China

Previous studies have shown that parenting is influenced by social norms and cultural values (Goodnow, 1985). Similar parenting styles may have different effects on children in different cultures (Leung et al., 1998). Therefore, it is very important to explore mindful parenting in line with Eastern culture. In China, there is a paucity of empirical research on mindful parenting though some research is beginning to emerge (Lo et al., 2018; Wang et al., 2018). It may be particularly relevant to explore this concept in China at it has the largest population of any country with its own specific culture and policies. For example, a one child policy was in place between 1980 and 2016. Thus, most of the teenagers now are an only child. Evidence shows that there are high levels of adolescent mental issues in adolescent children in China, such as anxiety, depression, and poor interpersonal relationships (Wu, 2012). Studies have shown that these mental health issues may be related to parenting style and parents’ psychological and behavioral control of their children (Xia and Liang, 2016; Gao et al., 2017). Exploring these parental factors in relation to mindful parenting and adolescent psychological morbidity (and well-being) in adolescents in China would be a potentially important endeavor. However, in order to do this, there is a need for a reliable and valid mindful parenting scale in Chinese. The few studies to be conducted in China have used the IM-P scale (Lo et al., 2018; Wang et al., 2018) which is useful though limited (see discussion above in Section “Mindful Parenting Scales”). Therefore, the aims of our study are to (1) translate the MIPQ into standard Mandarin and (2) explore the reliability and validity of this newly translated measure in a large sample of Chinese middle school parents.

### Convergent and Discriminant Validity and the MIPQ

Convergent validity means there should be a high correlation between different measures of the same construct. To some extent, the levels of individual mindfulness represents the levels of mindfulness one can bring into interpersonal relationships thus mindful parenting overlaps with individual mindfulness (Duncan et al., 2009). The Mindful Attention and Awareness Scale (MAAS) aims to measure individual mindfulness and has been correlated with the MIPQ to evaluate convergent validity of the MIPQ in studies of both the American (McCaffrey et al., 2017) and Turkish (Gördesli et al., 2018) versions of the MIPQ. Thus, we used this to test convergent validity in our study.

In addition, McCaffrey et al. (2017) used the Parental Authority Questionnaire (PAQ) and Parenting Scale (PS) to correlate with the MIPQ to examine convergent validity. Also, Gördesli et al. (2018), in a Turkish study, used the Parent-Children Communication Scale (PCCS; Kahraman, 2016) to correlate with the MIPQ to examine convergent validity. The PAQ, PS and PCCS are all measures related to parenting or parental behaviors. However, there are not Chinese versions of these three scales available. The Interpersonal Mindfulness in Parenting Scale (IM-P) and Parenting Styles & Dimensions Questionnaire (PSDQ), however, are two measures related to parenting which have Chinese versions. Therefore, we used the IM-P and PSDQ (alongside the MAAS) to correlate with the MIPQ to test its convergent validity. We expect that scores of the two dimensions of the MIPQ, mindful discipline and being in the moment with child, would be positively related to scores on the MAAS. We also predict that the IM-P and the authoritative parenting style in the PSDQ would be positively correlated with the MIPQ, while the authoritarian and permissive parenting style in the PSDQ would be negatively correlated.

Discriminant validity is the opposite of convergent validity, that is to say, there should be no (or a low) correlation between dissimilar constructs, or the correlation between dissimilar constructs should be negative. In the original validity testing of the MIPQ, socioeconomic status (SES) was used to test discriminant validity, with authors expecting no significant difference on MIPQ score among different levels of income, education and occupation of parents (McCaffrey et al., 2017). The results showed that MIPQ scores did not differ on any of the SES indicators apart from levels of income (higher income parents were more mindful parents). In addition, Kim et al. (2019) used depression, perceived stress, and pessimism to test the discriminate validity of the IM-P (Kim et al., 2019). They found that the IM-P was significantly negatively related to depression and perceived stress. Therefore, in line with these studies, our study used socioeconomic status (predicting no relation with MIPQ scores) and stress (Perceived Stress Scale) and depression (Beck’s Depression Inventory) measures (a negative correlation with MIPQ) to test discriminant validity for the MIPQ.

## Materials and Methods

### Participants

Participants were 1057 parents of students (12–16 years old) across two large middle schools in China. Participant demographics for the whole sample are shown in Table 1. The majority of the participants were mothers (68.7%), and ages ranged from 33 to 56 years. 45.9% of the participants lived in Tianjin, while 54.1% of the participants lived in Wuxi. The level of education of the sample was diverse, with 45.8% of the participants having completed a bachelor or junior college degree, 31.1% completed high school or a technical/vocational secondary school degree, and 23.1% completed middle school. The majority (58.3%) of parents were non-professional (e.g., manual workers or farmers) or unemployed; 28.1% of the sample were middle management or middle professional (e.g., teacher), while the remaining 13.6% worked in senior management or were a senior professional (e.g., company managers).

**TABLE 1:** Participant background and demographics (*n* = 1057).

	**Frequency**	**%**
**Place of residence**		
Tianjin	485	45.9
Wuxi	572	54.1
**Gender of child**		
Male	535	50.6
Female	522	49.4
**Gender of parents**		
Father	331	31.3
Mother	726	68.7
**Occupation of parents**		
Non-professional/unemployed	616	58.3
Middle management or middle professional	297	28.1
Senior management or senior professional	144	13.6
**Education of parents**		
No formal education or primary education	244	23.1
High school or technical secondary school	329	31.1
Bachelor degree or above	484	45.8
**Per capita income (in Chinese Yuan or RMB)**		
< ¥ 10000	114	10.8
10000∼40000	366	34.6
40000∼70000	349	33.0
>70000	228	21.6

### Procedure

It is essential to carry out translation and validation procedures prior to the application of an instrument in another population or culture. Thus, the study was conducted in two phases. Institutional review board approval was obtained prior to each phase. First, we translated the MIPQ into Standard Mandarin (Chinese) and back into English using the forward–backward method. Feedback on this newly translated measure was then gathered from a sample of middle school parents. Second, a large-scale validation study was conducted with middle school parents in order to test the psychometric properties (including validity and reliability) of this newly translated MIPQ.

#### Phase 1: Translation-Back Translation of the Mindfulness in Parenting Questionnaire (MIPQ)

We gained permission from the authors of the MIPQ to translate and adapt the scale (where necessary). There are 28 items in the original MIPQ, which has two dimensions, ‘being in the moment with the child’ and ‘mindful discipline.’ For each item, parents respond using a four-point rating scale (infrequently to almost always) to indicate whether each item is true for them over the past 2 weeks, with higher scores indicating greater levels of mindful parenting. Examples of items are: ‘Did you take time to listen and tune into your child when you two were talking?’ and ‘Did you ask your child’s opinion?’

One academic Psychology Lecturer and two postgraduate psychology students translated the original scale independently. They then discussed the accuracy and meaning of the translated content, until they reached an agreement. The key aspects they agreed to change (in line with Chinese language) were to: (1) change the questions to the first person (that is, instead of using ‘you’ changing to ‘I’) and (2) use declarative statements instead of questions. Thus, instead of ‘Did you carefully listen and tune into your child when you two were talking?’ The Chinese version of the statement is: ‘When I talk to my child, I listen carefully and respond to the child.’ Therefore, this latter change necessitated a change in the ordering of the sentence structure.

Following this, two postgraduates majoring in English who had never seen the original MIPQ back-translated the Chinese version separately. They then had a discussion on any discrepancies between their translations until they reached agreement. A panel including one postgraduate student of English, one Psychology Lecturer and one psychology postgraduate student compared the original version and back-translated version, discussed any inconsistencies and made necessary modifications where appropriate. Once they reached consensus, this was then piloted with ten parents of middle school students. We requested feedback on whether all items were easy to read and the meaning of each item was clear. There was one item that the parents found difficult to answer as they felt it was too vague and needed some context or parameters. Item 6 ‘Did you accurately predict in advance how your child would react to a situation’ was translated as ‘In certain situations, I can accurately predict the child’s reaction in advance.’ Parents were asked if the two items had the same meaning – they agreed it did, but the former item made it far easier to understand, interpret and give a score. After this modification, we then had a final Chinese draft of the scale (Chinese Mindful Parenting Questionnaire; C-MIPQ) to take forward to the next stage of the study (please see Table 2 for each item in Standard Mandarin and the item in the original English version that item was translated from).

**TABLE 2:** The Chinese MIPQ (English and Chinese items).

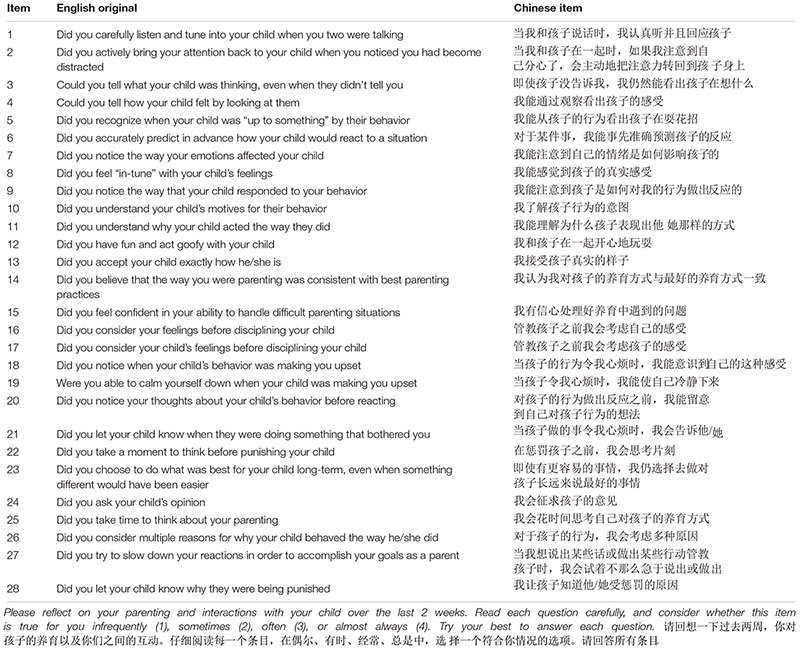

#### Phase 2 Validation of the Chinese Mindfulness in Parenting Questionnaire (C-MIPQ)

In China, students in middle school are aged between 12 and 16 years. As there can be differences in living environment, sub-culture, values and beliefs between southern China and northern China, we chose one middle school in the south and one in the north for data collection. Wuxi city and Tianjin city are representative cities in the south and north respectively thus to some extent participants are representative of the Chinese population. Many large-scale surveys in China have sampled in this way (e.g., Song et al., 2013; Li et al., 2016). The schools were chosen as they are local public schools with parents of similar SES status. Indeed, no difference across socioeconomic status variables between the parents in the two schools in our sample were found (occupation: χ 2 = 0.097, df = 2, *P* > 0.05; education: χ 2 = 0.742, df = 2, *P* > 0.05; income:χ 2 = 0.143, df = 3, *P* > 0.05).

We collected validation data in both schools across two waves of data collection (participants were from both schools across both waves of data collection though participants only took part once – with the exception of those who participated in the test-retest element). In the first wave of data collection participants across both schools (*n* = 662) completed the Chinese MIPQ and demographic information, with 121 completing the MIPQ 2 weeks later in order to examine test–retest reliability. In the second wave of data collection, different participants from the first wave of data collection (*n* = 395) completed a questionnaire booklet (see Materials and Methods) in order to test convergent and discriminant validity. Please see Table 3 for an overview of the two waves of data collection.

**TABLE 3:** Overview of the two waves of data collection.

**Data collection Wave 1**	**Data collection Wave 2**	**Overall Sample**
Tianjin city (*N* = 296)	Tianjin city (*N* = 189)	*N* = 1057
Wuxi city (*N* = 366)	Wuxi city (*N* = 206)	*N* = 1057
MIPQ *N* = 662	MIPQ *N* = 395	
SES/demographics *N* = 662	SES/demographics *N* = 395	
Retest MIPQ *N* = 121	IM-P *N* = 395	
	MAAS *N* = 395	
	CPSS *N* = 395	
	PSDQ *N* = 395	
	BDI-13 *N* = 395	

Participants were recruited in class meetings that all parents regularly attend. Two research assistants obtained informed written consent from participants. Once consent was established, survey packs were administered; the research assistants were present to answer questions as the parents completed the questionnaires.

##### Data collection 1: C-MIPQ completion and test–retest reliability

Seven hundred and fifty participants were eligible to take part in the first wave of data collection; 740 agreed to take part and 662 questionnaires could be used in the final analysis (with every section/item completed). Although not included in the original validity study by McCaffrey et al. (2017), assessment of test–retest reliability was considered useful in our study in order to evaluate whether the scale remains stable over time. Thus, at the end of the questionnaire booklet (which comprised the MIPQ and a demographic questionnaire – see Measures) participants were asked to indicate if they would be willing to complete another questionnaire after 2 weeks. From the 148 participants that were willing to receive the C-MIPQ again (for test–retest reliability) after 2 weeks, 121 (81.8%) of the participants completed these and sent them back. Thirty-eight of the 121 participants were male.

##### Data collection 2: MIQ completion and validity testing

Four hundred and twenty participants were eligible to take part in the second wave of data collection; 412 agreed to take part and 395 questionnaires could be used in the final analysis (with every section/item completed). The questionnaire pack for Sample 2 comprised seven questionnaires in order to test validity (see Measures); a brief demographic questionnaire, the Mindful Attention and Awareness Scale (MAAS), the Chinese Perceived Stress Scale (CPSS), Parenting Styles and Dimensions Questionnaire-Short Versions (PSDQ-short), the Beck Depression Inventory (BDI-13), the Interpersonal Mindfulness in Parenting Scale (IM-P) as well as the new Chinese MIPQ (C-MIPQ).

### Measures

#### Demographic Questionnaire

Participants completed a brief demographic questionnaire that included information regarding the parents’ age, gender, child’s age and socioeconomic status (education, employment status and income).

#### Mindful Attention and Awareness Scale (MAAS; Deng et al., 2012)

In order to assess convergent validity of the C-MIPQ, the Mindful Attention and Awareness Scale (MAAS) was included in the questionnaire pack. This questionnaire was originally developed in English (Brown and Ryan, 2003) and is a 15-item unidimensional measure of intrapersonal mindfulness. It has since been translated and validated in a Chinese sample (Deng et al., 2012) and used to assess intrapersonal mindfulness in Chinese adults in several studies (Zhao et al., 2016; Fang et al., 2018; Lei et al., 2018). Participants respond to items using a six-point Likert-type rating scale, from 1 to 6 indicating ‘almost always’ to ‘almost never.’ Example items include ‘I find it difficult to stay focused on what is happening in the present’ and ‘It seems I am “running on automatic,” without much awareness of what I am doing.’

The total score of the scale ranges from 15 to 90. The higher the score, the higher the individual’s mindfulness level. In the present study, the Cronbach’s alpha coefficient of the scale was 0.84.

#### Chinese Mindfulness in Parenting Questionnaire (C-MIPQ)

As outlined in the Procedure the C-MIPQ comprised 28 items (like the original English version). For each item, parents respond using a five-point Likert-type rating scale (never to almost always) to indicate whether each item is true for them over the past 2 weeks.

#### Chinese Perceived Stress Scale (CPSS; Yang and Huang, 2003)

The Perceived Stress Scale (CPSS), was originally developed by Cohen et al. (1983) and Yang and Huang (2003) translated it into Chinese. The scale comprises 14 items with two subscales which are ‘Out of control’ (example item ‘How often in the last month were you unable to control the important things in your life?’) and ‘Tension’ (example item ‘In the last month, how often have you felt nervous and stressed?). It is scored on a 5-point scale (ranging from never to very often) and the total score can range between 14 and 70. Higher scores indicate greater perceived stress. In the present study, the Cronbach’s alpha coefficient of the CPSS was 0.78.

#### Chinese Parenting Styles and Dimensions Questionnaire-Short Version (PSDQ-Short Version; Yan et al., 2016)

The Parenting Styles and Dimensions Questionnaire-Short Version (Robinson et al., 2001) is a 32 item scale that measures parenting patterns. Participants respond on a five-point Likert scale (1 = never; 5 = always). The scale includes three dimensions of parenting: authoritarian, authoritative and permissive parenting. Only sub-scale scores can be used. We used the Chinese version translated and validated by Yan et al. (2016).

#### Beck Depression Scale (BDI-13; Zhang and He, 2015)

We used the Chinese version (Zhang and He, 2015) of the 13-item Beck Depression Scale (BDI-13; Beck et al., 1996). It comprises 13 items, in which four response options are presented on a scale of 0–3. For example, “I don’t feel depressed” (score of 0) to “I’m so depressed that I can’t stand it anymore” (score of 3). A higher score indicates higher depression. The Cronbach’s alpha coefficient for this scale in this study was 0.84.

#### Chinese Interpersonal Mindfulness in Parenting Scale (IM -P; Lo et al., 2018)

The Interpersonal Mindfulness in Parenting scale (IM-P) was developed by Duncan et al. (2009) and translated into Chinese by Lo et al. (2018). It comprises 23 items, with a five-point Likert response scale (from never to always). The scale includes four dimensions: compassion for the child (e.g., ‘I can maintain patience with my children when I’m struggling’), non-judgmental acceptance in parenting (e.g., ‘As a parent, I don’t criticize myself’), emotional awareness in parenting (e.g., ‘When I’m not happy with my children, I try to maintain emotional stability’) and listening with full attention (e.g., ‘I listen to my child with full attention’). The Cronbach’s alpha coefficient for this scale in our study was 0.83.

### Data Analyses

Evaluation of the MIPQ items was a multi-step process that included evaluation of (a) item-total correlation analysis, (b) reliability (internal consistency and test-retest reliability) (c) construct validity (d) convergent validity (e) discriminant validity and (f) group invariance.

Item-total correlations were calculated by using Pearson’s Product Moment correlations to correlate each item of the scale with the total score of the scale. These represent the extent to which items measure the same construct as the other items (corrected total scale). We evaluated the internal consistency of the C-MIPQ using Cronbach’s alpha, which reflects the overall correlation between items within a scale. Test-retest reliability was established using the intraclass correlation coefficient between Time 1 and Time 2 for the sub-set of participants who completed the C-MIPQ 2 weeks apart. Construct validity was established by conducting a confirmatory factor analysis based on the two-factor solution found in the original MIPQ validation study. The convergent validity of the scale was assessed by using a Pearson’s product moment correlation to correlate the C-MIPQ with a number of different related measures (the MAAS, the IM-P and the PSDQ). Discriminant validity was assessed by conducting an ANOVA to establish whether there was a difference in C-MIPQ scores for the SES variables (income, education and occupation). Moreover, Pearson’s product moment correlation was used to establish whether there was a negative relationship between the C-MIPQ and perceived stress (CPSS) and depression (BDI-13). Group invariance was tested for mothers and fathers on MIPQ scores and also across the two schools in terms of MIPQ scores. This was done by defining the categories first then analyzed using the configuration model and the weak measurement invariance test.

Analyses were conducted in AMOS version 24.0 and SPSS version 22.0. A significance level of alpha = 0.05 was used.

## Results

### Item Analysis

A correlation between each item in the scale and the total score of the scale was calculated (see Table 4). Results showed that the correlation coefficient between each of the 28 items and the total score of the scale ranged from 0.49 to 0.72, and all the correlation coefficients reached a significance level of *p* < 0.01.

**TABLE 4:** Mean (standard deviation) scores, skewness/kurtosis and corrected item-total correlation for the Chinese MIPQ (*n* = 1057).

**Items**	**Mean (*SD*)**	**Range**	**Corrected item-total correlation**	**Skewness**	**Kurtosis**
**Factor 1: Mindful discipline**					
Item 14	2.57 (0.87)	1–5	0.55	0.45	2.03
Item 15	2.80 (0.84)	1–5	0.59	–0.25	–0.56
Item 16	2.49 (0.87)	1–5	0.50	0.06	–0.66
Item 17	2.85 (0.79)	1–5	0.61	–0.22	–0.48
Item 18	2.94 (0.82)	1–5	0.55	0.41	4.99
Item 19	2.54 (0.83)	1–5	0.61	0.01	–0.54
Item 20	2.68 (0.78)	1–5	0.72	–0.13	–0.39
Item 21	2.98 (0.79)	1–5	0.53	–0.33	–0.51
Item 22	2.73 (0.88)	1–5	0.58	–0.19	–0.70
Item 23	3.13 (0.77)	1–5	0.56	–0.51	–0.34
Item 24	3.15 (0.73)	1–5	0.64	–0.52	–0.15
Item 25	2.96 (0.79)	1–5	0.63	–0.38	–0.34
Item 26	2.99 (0.73)	1–5	0.64	–0.33	–0.21
Item 27	2.60 (0.78)	1–5	0.58	0.03	–0.42
Item 28	3.34 (0.71)	1–5	0.57	–0.91	0.52
Cronbach’s α = 0.89					
**Factor 2: Being in the moment with the child**					
Item 1	3.30 (0.70)	1–5	0.56	–0.83	0.67
Item 2	2.99 (0.88)	1–5	0.55	–0.47	–0.59
Item 3	2.77 (0.84)	1–5	0.54	0.23	2.00
Item 4	3.06 (0.77)	1–5	0.64	–0.49	–0.20
Item 5	2.98(0.84)	1–5	0.49	–0.51	–0.34
Item 6	2.81 (0.78)	1–5	0.59	–0.30	–0.26
Item 7	2.85 (0.82)	1–5	0.58	–0.31	–0.46
Item 8	2.91 (0.78)	1–5	0.66	–0.30	–0.06
Item 9	2.98 (0.76)	1–5	0.59	–0.42	–0.13
Item 10	2.93 (0.78)	1–5	0.65	–0.41	–0.15
Item 11	2.83 (0.81)	1–5	0.63	–0.34	–0.34
Item 12	2.87 (0.86)	1–5	0.55	–0.25	–0.75
Item 13	3.25 (0.80)	1–5	0.57	–0.84	0.07
Cronbach’s α = 0.88					

### Reliability Analysis

The Cronbach’s alpha coefficient of the C-MIPQ was 0.93, demonstrating excellent internal consistency; the Cronbach’s alpha coefficient of Factor 1 was 0.89, and Factor 2 was 0.88. The test-retest intra-class correlation of the MIPQ was 0.83, and the retest reliability of Factor 1 and Factor 2 were both 0.80.

### Validity Analysis

#### Construct Validity (Confirmatory Factor Analysis)

Confirmatory factor analysis tests specific theoretical models based on our previous knowledge of inter-relationships between observed variables. A two-factor model was found by the authors of the MIPQ (McCaffrey et al., 2017). Thus, in order to test the fit of this suggested underlying model for the Chinese MIPQ, a two-factor model was constructed. The goodness-of-fit of the model was evaluated using multiple criteria (see Table 5), the value of χ 2/df was less than 3 (2.95). The fitting indices such as CFI, RFI, GFI, NFI, and IFI were all greater than 0.85, and the RMSEA value was less than 0.08 (0.04), indicating that the two-factor model fits well with the observed data (Wen et al., 2004).

**TABLE 5:** Confirmatory factor analysis model fitting index for the Chinese version of the MIPQ.

	**χ^2^/df**	**GFI**	**NFI**	**CFI**	**RFI**	**IFI**	**RMSEA**
Two-factor	2.95	0.94	0.92	0.95	0.90	0.95	0.04

**FIGURE 1:**
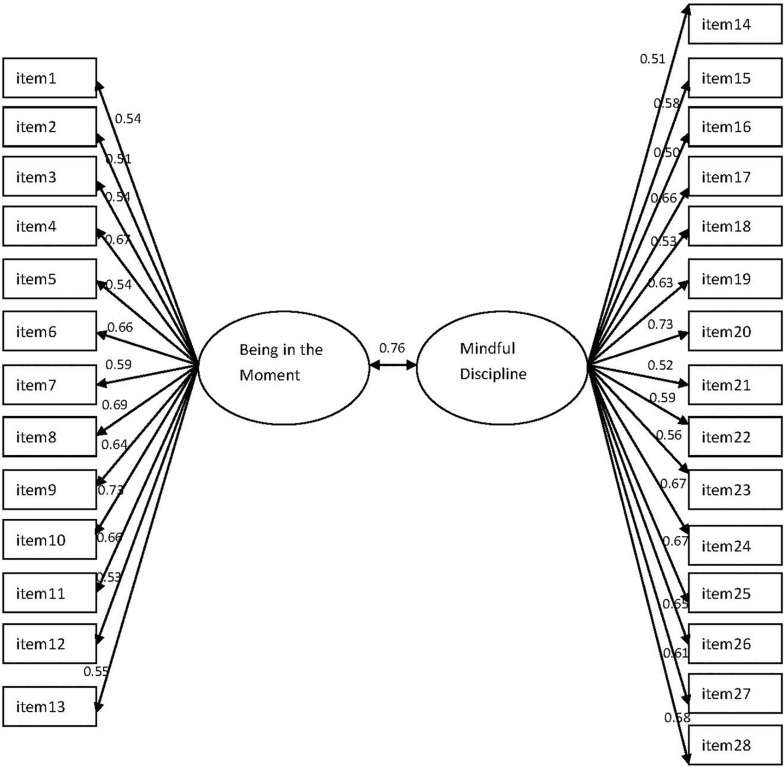
Confirmatory factor analysis of the MIPQ two-factor model.

As with the English version of the MIPQ, items comprising Factor 1 (15 items) were parent-focused, and content reflected non-reactivity in parenting, parenting awareness, and goal-focused parenting (‘mindful discipline’). Factor 2 (13 items) appeared to represent a child-focused facet of mindful parenting, which included present-centered attention, empathy and acceptance of the child – ‘being in the moment with the child.’ See Figure 1. The correlation coefficients of Factor 1 and Factor 2 and the total score of the MIPQ were both 0.93 (*p* < 0.001).

#### Convergent Validity

For the mean and standard deviation for all of the scales used for validity purposes see Table 6. In terms of convergent validity, it was hypothesized that both MIPQ factors would be positively related to interpersonal mindfulness as measured by the MAAS. This hypothesis was supported (see Table 7). To further establish convergent validity, it was also expected that the two MIPQ factors would be positively related to parents’ compassion for their child, listening with full awareness, non-judgmental acceptance in parenting and emotional awareness in parenting measured by IM-P. This was also supported (see Table 7).

**TABLE 6:** Mean (standard deviation) for scales used to establish validity.

**Scales and subscales (number of items)**	**Minimum**	**Maximum**	***M***	***SD***
BDI-13 (13)	6	30	17.73	4.24
MAAS (15)	29	90	72.43	10.34
IM-P overall score (23)	58	95	76.59	6.71
IM-P: CC (7)	16	35	27.00	3.86
IM-P: NJAP (6)	6	30	17.73	4.24
IM-P: EAP (6)	12	30	21.03	3.10
IM-P: LFA(4)	6	20	13.27	2.35
PSDQ: Authoritative (14)	19	75	54.44	9.89
PSDQ: Authoritarian (12)	12	60	24.77	7.56
PSDQ: Permissive (5)	2	25	12.08	3.43
CPSS overall score (14)	20	60	36.24	6.54
CPSS: Out of Control (7)	7	35	17.53	4.58
CPSS: Tension (7)	7	31	18.45	4.02

*MAAS, Mindful Attention and Awareness Scale; IM-P: CC, the subscale compassion for their child in the Interpersonal Mindfulness in Parenting Scale; IM-P: NJAP, non-judgmental acceptance in parenting subscale of the Interpersonal Mindfulness in Parenting Scale; IM-P: EAP, emotional awareness in parenting subscale in the Interpersonal Mindfulness in Parenting Scale; IM-P: LFA, the subscale listening with full attention of Interpersonal Mindfulness in Parenting Scale; PSDQ Authoritative, the authoritative subscale of Parenting Styles and Dimensions Questionnaire-Short Version; PSDQ Authoritarian, the authoritarian in the Parenting Styles and Dimensions Questionnaire-Short Version; PSDQ Permissive, the permissive subscale of the Parenting Styles and Dimensions Questionnaire-Short Version.*

**TABLE 7:** Correlations of the MIPQ with associated measures to establish convergent validity.

**Measure**	**Factor 1:**		**Factor 2: being in the**	
	**mindful discipline**		**moment with the child**	
	***r***	***(p)***	***r***	***(p)***
MAAS	0.42	0.000^∗∗∗^	0.46	0.000^∗∗∗^
IM-P: CC	0.80	0.000^∗∗∗^	0.65	0.000^∗∗∗^
IM-P: NJAP	0.43	0.000^∗∗∗^	0.33	0.000^∗∗∗^
IM-P: EAP	0.58	0.000^∗∗∗^	0.45	0.000^∗∗∗^
IM-P: LFA	0.37	0.000^∗∗∗^	0.31	0.000^∗∗∗^
PSDQ: Authoritative	0.54	0.000^∗∗∗^	0.51	0.000^∗∗∗^
PSDQ: Authoritarian	–0.34	0.000^∗∗∗^	–0.21	0.000^∗∗∗^
PSDQ: Permissive	–0.39	0.000^∗∗∗^	–0.31	0.000^∗∗∗^

^*^p < 0.05; ^∗∗^*p* < 0.01; ^∗∗∗^*p* < 0.001. MAAS, Mindful Attention and Awareness Scale; IM-P: CC, the subscale compassion for their child in the Interpersonal Mindfulness in Parenting Scale; IM-P: NJAP, non-judgmental acceptance in parenting subscale of the Interpersonal Mindfulness in Parenting Scale; IM-P: EAP, emotional awareness in parenting subscale in the Interpersonal Mindfulness in Parenting Scale; IM-P: LFA, the subscale listening with full attention of Interpersonal Mindfulness in Parenting Scale; PSDQ Authoritative, the authoritative subscale of Parenting Styles and Dimensions Questionnaire-Short Version; PSDQ Authoritarian, the authoritarian in the Parenting Styles and Dimensions Questionnaire-Short Version; PSDQ Permissive, the permissive subscale of the Parenting Styles and Dimensions Questionnaire-Short Version.

We hypothesized that the two factors on the MIPQ would be positively related to an authoritative parenting style, and negatively related to authoritarian and permissive parenting styles, as measured by the PSDQ. Results also supported this hypothesis (Table 7).

#### Discriminant Validity

Next, discriminant validity of the MIPQ was evaluated. To establish discriminant validity, it was hypothesized that mindful parenting would be significantly negatively related to the parent’s depression measured by the BDI-13 and the subscales ‘Out of Control’ and ‘Tension’ measured by the CPSS. A significant negative relationship was found between the two factors on the MIPQ and the BDI-13 (factor 1 *r* = −0.42, *p* < 0.001; factor 2 *r* = −0.31, *p* < 0.001). Both mindful discipline and being in the moment with the child were significantly negatively related to feeling Out of Control (factor 1 *r* = −0.44; *p* < 0.001; factor 2 *r* = −0.41, *p* < 0.001) and Tension (factor 1 *r* = −0.39, *p* < 0.001; factor 2 *r* = −0.31, *p* < 0.001) on the CPSS.

We further evaluated the discriminant validity of the MIPQ by testing the hypothesis that mindful parenting would not differ according to socioeconomic status variables (employment status, educational attainment, and household income). Results of the ANOVA showed that both ‘mindful discipline’ and ‘being in the moment with the child’ scores were not significantly different depending on employment status (factor 1 *F* = 1.73, *p* = 0.178; factor 2 *F* = 2.77, *p* = 0.063). Moreover, there was no difference in MIPQ scores for either factor depending on level of income (factor 1 *F* = 1.75, *p* = 0.16; factor 2 *F* = 0.38, *p* = 0.770). ‘Mindful discipline’ (Factor 1) did not vary across levels of education (*F* = 1.28, *p* = 0.280) however it did on the ‘being in the moment with the child’ MIPQ subscale (Factor 2) (*F* = 9.69, *p* < 0.001). *Post hoc* analyses revealed that parents who reported a degree of junior high school level or below had significantly lower ‘in the moment with the child’ scores than parents who had a bachelor degree or higher qualification (*p* = 0.006).

#### Group Invariance

We explored the group invariance between fathers and mothers on C-MIPQ scores. The results showed there was group invariance between fathers and mothers (Δχ^2^ = 25.969, df = 18, *p* > 0.05; ΔCFI < 0.01). We also explored the group invariance between the two schools. The results showed there was group invariance in C-MIPQ scores between the two schools (Δχ 2 = 50.908, df = 54, *p* > 0.05; ΔCFI < 0.01).

## Discussion

The aims of our study were to (1) translate the MIPQ into standard Mandarin and (2) explore the reliability and validity of this newly translated measure in a large sample of Chinese middle school parents. As far as we are aware, this is the first study to translate and provide preliminary data on validity on this questionnaire in Chinese.

The preliminary developmental work we conducted helped ensure that the MIPQ was structured in a manner that made sense in Standard Mandarin and was culturally meaningful for Chinese parents. In order to do this, we used the forward–backward method of translation and included a total of five independent translators (both those from a Psychology background for the forward translation, those without a Psychology background in the back-translation and a mix of both for the final panel). In addition, the draft C-MIPQ was given to a pool of parents to evaluate ease of reading and meaning/understanding. These steps resulted in what we believe to be a semantically equivalent scale to the original. Although we changed items to the first person (‘I’ instead of ‘You’) and framed these as a declarative statement (instead of a question), this did not change the actual meaning of the items. Item 6 was changed from ‘Did you accurately predict in advance how your child would react to a situation?’ to ‘In certain situations, I can accurately predict the child’s reaction in advance’ from feedback from the parents in Phase 1. We checked with these same ten parents and they claimed they would answer the same way for both items, but the latter construction made more sense in Chinese. Therefore, we feel we remained faithful to the original scale, and did not need to remove or significantly alter items for cultural appropriateness/relevance.

The internal consistency of the C-MIPQ was determined using Cronbach’s alpha. For psychometric scales Cronbach’s α > 0.8 is generally recommended (Streiner and Norman, 2003). Thus, this scale demonstrated a high level of internal consistency (α = 0.93; each subscale α = 0.80). Inter-item correlations were moderate to strong. The test-retest intra-class correlation demonstrated that the C-MIPQ is stable over time (in this instance across a 2-week period). This is a novel finding as test-retest reliability was not included in the original validation study of the MIPQ (McCaffrey et al., 2017). Thus, mindful parenting, as measured by the C-MIPQ, appears to be a regular, or consistent, aspect of parenting.

Confirmatory factor analysis was performed to test whether our data fit the two-factor model found with the original version of the MIPQ. The results of the CFA show that the two-factor model was a good fit. Regarding convergent validity, it was hypothesized that the MIPQ (total score and also both factors) would be distinct, but positively related (a moderate positive correlation) to interpersonal mindfulness as measured by the Mindful Attention and Awareness Scale (MAAS). As we expected, there was a moderate significant correlation between the overall MIPQ score and the MAAS and both MIPQ factors and the MAAS. This indicates that intrapersonal and interpersonal mindfulness are related but distinct constructs. This mirrors McCaffrey et al.’s (2017) study where they also found significant correlations between the MIPQ and MAAS (as an indicator of convergent validity) though the original version of the scale had a weak-medium correlation.

We hypothesized that the two factors on the MIPQ would be positively related to an authoritative parenting style, and negatively related to authoritarian and permissive parenting styles, as measured by the PSDQ. Our results supported this hypothesis, providing further evidence of convergent validity. Geurtzen et al. (2015) also found that parents with high levels of mindful parenting encourage their child to be autonomous and self-manage their own behavior, with less use of psychological control.

As a further test of convergent validity, we predicted that the IM-P would be positively correlated with the C-MIPQ as they are both measures of mindful parenting. We did indeed find that all sub-scales on the IM-P significantly correlated with both factors on the C-MIPQ, showing support for convergent validity. Although the IM-P is a measure of mindful parenting there are some limits to its use. The IM-P is only applicable to adolescents aged 10–14 and is restricted to mothers. Moreover, some of the subscales of the IM-P overlap with one another and some sub-scales only include items from either a parent-oriented or child-oriented facet. Thus, we consider the MIPQ the scale of choice when assessing mindful parenting.

In order to assess the discriminant validity of the C-MIPQ, parents’ stress levels and depression status were correlated with scores on the C-MIPQ. As predicted, mindful parenting was negatively related to stress and depression. Previous research has shown that parents with higher levels of mindful parenting have lower levels of parenting pressure and less negative emotions (Parent et al., 2010; Geurtzen et al., 2015; Tak et al., 2015; Corthorn and Milicic, 2016). High levels of mindful parenting can help parents be more aware of their children’s emotions, and then adjust their parenting behaviors accordingly (Townshend et al., 2016). This can help develop a good parent-child relationship, which may reduce parental parenting pressure.

We further evaluated the discriminant validity of the MIPQ by testing the hypothesis that mindful parenting would not differ across different socioeconomic status. Results showed that scores of the two subscales ‘mindful discipline’ and ‘being in the moment with the child’ were not significantly different among different employment status or levels of income. ‘Mindful discipline’ scores did not vary across levels of parents’ education, while ‘being in the moment with the child’ scores did. Parents with undergraduate or postgraduate degrees had higher level of being in moment with the child than those with high school or junior high school as their highest educational attainment. This may be because more highly educated parents are more perceptive in terms of what their child is saying and respond to the child’s needs and emotions in the moment, and thus focus more on ‘being present’ (Duncan et al., 2009).

The limitations and strengths of the study should be acknowledged. First, although we sampled from two different cities/provinces (Tianjin in the North of China and Wu Xi in Jiang Su Province in the South of China) we cannot purport to generalize our findings across the whole of China. Future researchers may want to sample from a wider range of provinces across different demographic groups. In addition, we were specifically interested in adolescents (12–16 years old) therefore our sample comprised only this age group. If the C-MIPQ was to be used in China with a younger sample (and the original version is validated with parents whose children’s ages from of 2–16 years; McCaffrey et al., 2017) it would need to be tested and validated across relevant age groups first. In terms of strengths, McCaffrey and colleagues acknowledge in their original validation study in the USA that a larger sample size would have been beneficial – we feel we achieved this (1057 participants), with most of the participants approached willing to participate in the study.

## Conclusion

Our study is the first step toward establishing the psychometric properties of the Mindfulness in Parenting Questionnaire in Chinese (C-MIPQ) (Appendix A1) for parents of adolescents aged 12–16 years. Further studies will be needed in order to test this further. Researchers who want to explore mindful parenting in parents of young children may want to use this measure as a starting point when testing and validating it with parents of younger children.

This C-MIPQ should help facilitate mindful parenting research in China, a research area which is currently under-developed. Within this, it will allow researchers to assess mindful parenting in relation to key variables such as interpersonal mindfulness, parenting styles, behaviors and parental psychopathology in order to understand the association between parental factors and child well-being and psychological distress in Chinese adolescents. This may also include cross-cultural studies and testing the effectiveness of interventions to facilitate mindful parenting.

## Data Availability

The datasets generated for this study are available on request to the corresponding author.

## Ethics Statement

This study was carried out in accordance with the recommendations of the Institutional Review Board of Tianjin Normal University and written consent was gained from all participants. The protocol was approved by the Institutional Review Board of Tianjin Normal University, China.

## Author Contributions

LW, HB, and YZ made important contributions in the writing of this manuscript. HB was in charge of modifying the manuscript. LW led the writing work and took the responsibility as first author. YZ and ZZ analyzed the data. PW, ZZ, BZ, and BF helped to collect the data.

## Conflict of Interest Statement

The authors declare that the research was conducted in the absence of any commercial or financial relationships that could be construed as a potential conflict of interest.

## APPENDIX A1

**Table:** The Chinese version of the Mindfulness in Parenting Questionnaire (MIPQ)
